# Delayed angiopoietin‐2 blockade reduces influenza‐induced lung injury and improves survival in mice

**DOI:** 10.14814/phy2.15081

**Published:** 2021-11-09

**Authors:** Jeffrey E. Gotts, Mazharul Maishan, Lauren Chun, Xiaohui Fang, Chun‐Ya Han, Venice Chiueh, Aarif Y. Khakoo, TaeWeon Lee, Marina Stolina, Michael A. Matthay

**Affiliations:** ^1^ Departments of Medicine and Anesthesia Cardiovascular Research Institute University of California, San Francisco San Francisco California USA; ^2^ Department of Cardiometabolic Disorders Amgen Research Thousand Oaks California USA

**Keywords:** angiopoietin‐2, influenza, pneumonia, pulmonary edema, viral lung injury

## Abstract

Influenza remains a major cause of death and disability with limited treatment options. Studies of acute lung injury have identified angiopoietin‐2 (Ang‐2) as a key prognostic marker and a potential mediator of Acute respiratory distress syndrome. However, the role of Ang‐2 in viral pneumonia remains poorly defined. This study characterized the time course of lung Ang‐2 expression in severe influenza pneumonia and tested the therapeutic potential of Ang‐2 inhibition. We inoculated adult mice with influenza A (PR8 strain) and measured angiopoietin‐1 (Ang‐1), Ang‐2, and Tie2 expressions during the evolution of inflammatory lung injury over the first 7 days post‐infection (dpi). We tested a peptide‐antibody inhibitor of Ang‐2, L1‐7, administered at 2, 4, and 6 dpi and measured arterial oxygen saturation, survival, pulmonary edema, inflammatory cytokines, and viral load. Finally, we infected primary human alveolar type II epithelial (AT2) cells grown in air‐liquid interface culture with influenza and measured Ang‐2 RNA expression. Influenza caused severe lung injury between 5 and 7 dpi in association with increased Ang‐2 lung RNA and a dramatic increase in Ang‐2 protein in bronchoalveolar lavage. Inhibition of Ang‐2 improved oxygenation and survival and reduced pulmonary edema and alveolar‐capillary barrier permeability to protein without major effects on inflammation or viral load. Finally, influenza increased the expression of Ang‐2 RNA in human AT2 cells. The increased Ang‐2 levels in the airspaces during severe influenza pneumonia and the improvement in clinically relevant outcomes after Ang‐2 antagonism suggest that the Ang‐1/Ang‐2 Tie‐2 signaling axis is a promising therapeutic target in influenza and potentially other causes of viral pneumonia.

## Graphical abstract

Angiopoietin‐2 (Ang‐2) is a key prognostic marker and potential mediator of Acute respiratory distress syndrome, yet it is unknown whether inhibiting Ang‐2 signaling at its receptor Tie‐2 can reduce viral lung injury after it begins. Here we demonstrate that delayed antagonism of Ang‐2 in mice with severe influenza pneumonia improves survival, gas exchange, and pulmonary edema.

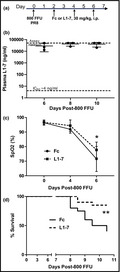

## 1 INTRODUCTION

Acute respiratory distress syndrome (ARDS) remains a major cause of morbidity and mortality, responsible for approximately 75,000 deaths annually in the United States (Rubenfeld et al., 2005). Historically, influenza has been the major viral etiology of ARDS, causing widespread seasonal epidemics and occasional pandemics as in 2009. Influenza may be prevented by vaccination, but once an infection is established available therapies such as neuraminidase inhibitors have limited benefit (Dobson et al., 2015; Jefferson et al., 2014). More recently, the novel human coronavirus syndromes SARS, MERS, and now COVID‐19 have emerged as serious threats to public health; no disease‐modifying therapies have yet been approved for the treatment of ARDS caused by these viruses. There is a compelling need to develop therapeutics that improve clinically important outcomes in viral pneumonia when administered several days following established infection.

The angiopoietin‐1 (Ang‐1) Tie‐2 axis has emerged as an important mechanistic pathway in the development of acute lung injury (Parikh, 2013). During health, Ang‐1 is secreted by perivascular cells and platelets. Ang‐1 binds the Tie‐2 receptor on endothelial cells, resulting in its cytoplasmic phosphorylation and downstream signaling through PI3‐kinase/AKT and ERK, maintaining low lung vascular permeability (Thurston & Daly, 2012). Angiopoietin‐2 (Ang‐2), stored in endothelial Weibel‐Palade bodies, is released in response to inflammatory stimuli, antagonizing the action of Ang‐1 at Tie‐2 and resulting in increased vascular permeability. Specific genetic variants in Ang‐2 signaling pathways have been found to increase the risk of ARDS (Meyer et al., 2011; Reilly et al., 2018), whereas the concentration of Ang‐2 in the plasma of critically ill patients in the emergency department predicts its subsequent development (Agrawal et al., 2013). Finally, plasma Ang‐2 has been shown by multiple groups to independently predict ARDS mortality in both children (Yehya et al., 2016; Zinter et al., 2016) and adults (Li et al., 2020).

In experimental models, systemic administration of Ang‐2 is sufficient to increase lung endothelial permeability (Parikh et al., 2006). Furthermore, Tie‐2 agonism by Ang‐1‐expressing adenovirus or the Ang‐1 peptide mimetic vasculotide has been shown to improve experimental lung injury caused by endotoxin (David et al., 2011; Huang et al., 2008), hemorrhagic shock (Trieu et al., 2018), cardiopulmonary bypass (Dekker et al., 2018), and pneumococcal pneumonia (Gutbier et al., 2017). Vasculotide has also been shown to reduce the severity of lung injury in influenza pneumonia (Sugiyama et al., 2015). However, the temporal expression patterns of Ang‐1 and Ang‐2 during influenza pneumonia remain unclear, as does the therapeutic potential of directly targeting Ang‐2. Here we report that severe influenza infection in mice causes a significant increase in airspace Ang‐2 between 5 and 7 days post‐infection (dpi), and that delayed administration of the Ang‐2 inhibitor L1‐7 improves oxygenation, pulmonary edema, and survival.

## 2 MATERIALS AND METHODS

### 2.1 Animals, viral infection, and pulse oximetry

Adult (8–12 weeks old) C57BL/6 mice were ordered from NCI, housed in pathogen‐free housing, and cared for in accord with NIH guidelines by the Laboratory Animal Resource Center of the University of California, San Francisco (UCSF). All experiments were conducted under protocols approved by the UCSF Institutional Animal Care and Use Committee. Group size was determined to ensure adequate statistical power based on our extensive experience with models of acute lung injury (Fang et al., 2015; Gotts et al., 2017; Lee et al., 2011). Mice were deeply anesthetized with isoflurane, and between 100 and 800 foci‐forming units of Influenza A/H1N1/Puerto Rico/8/34 (PR8) dissolved in 30 µl of PBS was administered by nasal inoculation, as in our prior work (Gotts et al., 2014). Mice were weighed daily, and pulse oximetry was measured using the MouseOx+ cervical collar system (Starr Life Sciences), and as in our prior studies, the mean SpO_2_ during 5 min of recording was calculated (Gotts et al., 2017, 2018).

### 2.2 L1‐7 treatment

The peptide‐Fc fusion protein L1‐7 (MW 58.3 kDa) shows a high specificity for competitive inhibition of the Ang‐2‐Tie2 interaction, with an IC_50_ of 0.054 nM in humans and 0.071 nM in mice (Oliner et al., 2004). In uninjured mice, L1‐7 has a half‐life of 56 h (Oliner et al., 2004). L1‐7 has been shown to potently inhibit VEGF‐induced corneal angiogenesis (Oliner et al., 2004), and to normalize implanted human colorectal tumor blood vessels in mice (Falcón et al., 2009).

### 2.3 Lung injury endpoints

For wet‐dry ratio, mice were killed by bilateral thoracotomy after overdose of ketamine, blood was collected by RV puncture, and the lungs were homogenized in 1 ml PBS. Samples of blood, lung homogenate, and homogenate supernatant were weighed before and after desiccation, and another fraction of homogenate was assayed for hemoglobin concentration such that the blood volume of the lung and wet‐dry ratio could be calculated (Su et al., 2007). In separate animals, bronchoalveolar lavage (BAL) was accomplished by tracheal cannulation and lavage with two 250 μl aliquots of PBS. Histological analysis was performed on the lavaged mice. Lungs were fixed by intratracheal installation of 1 ml 4% paraformaldehyde followed by overnight fixation, dehydration, paraffin embedding, and staining of 4 µm sections with hematoxylin and eosin.

### 2.4 Measurement of protein biomarkers of inflammation and lung injury

Systemic (serum) and lung (BAL fluid) concentrations of the pro‐inflammatory cytokines IL‐1α, IL‐1β, IL‐6, KC (murine homologue of IL‐8), TNF‐α, IFN‐γ, and MCP‐1 were evaluated using the Mouse Premix Panel plex (R&D Systems). Commercial ELISA kits were used for the detection of mouse Ang‐1 (MyBioSource), Ang‐2 (R&D Systems). All assays were performed in accordance with manufacturer protocols.

### 2.5 Measurement of mRNA expression

Left lung lobes were isolated and flash‐frozen in liquid nitrogen at necropsy, and pulverized using stainless steel Bessman Tissue Pulverizers (Cole‐Parmer). Tissue powders were homogenized and prepared according to the Quantigene Sample Processing Kit protocol (Affymetrix). Extracts were tested for RNA presence and quality using a Bioanalyzer 2100 (Agilent Technologies). The mRNA expression levels of *angpt1*, *angpt2*, and *CD31* were evaluated relative to that of the housekeeping gene *hprt1* using Quantigene Plex 2.0 kits (Affymetrix) in accordance with manufacturer protocols.

### 2.6 Influenza viral load measurements

At 7 dpi, mice were killed and the left lung was placed in RNA Shield (Zymo Research), incubated at 4°C overnight, and then frozen at −20°C. Samples were thawed, minced with a scalpel, then homogenized, and extracted using a Zymo Quick viral RNA kit (Zymo Research). Extracts were tested for RNA presence and quality using a DS‐11 Fx+ spectrophotometer (DeNovix), and cDNA was created using a High Capacity cDNA Reverse Transcription Kit (Applied Biosystems). RT‐PCR was performed using a SYBR Green Kit (Bio‐Rad) and a LightCycler (Roche) with primers specific for influenza viral nucleoprotein A (forward: CAGCCTAATCAGACCAAATG, backward: TACCTGCTTCTCAGTTCAAG) as in Kim et al. (2014), and murine GAPDH (forward: AAGGTCATCCCAGAGCTGAA, backward: CTGCTTCACCACCTTCTTGA). The concentration of mRNA specific for VNP to that of GAPDH was then calculated.

### 2.7 Human AT2 cell isolation and culture

Alveolar type II epithelial (AT2) cells used in these experiments were isolated from the right middle lobe of a 56‐year‐old female non‐smoker whose lungs were declined for transplantation by the Northern California Transplant Donor Network according to our well‐established protocols (Fang et al., 2006, 2010; Lee et al., 2007). The isolated AT2 cells were seeded at a density of 1 × 10^6^ cells/well on collagen I‐coated 24‐well Transwell (3495, Costar). The cells were cultured in a 37°C and 5% CO_2_ incubator in DMEM high glucose 50%, F‐12 50% mix medium containing 10% FBS and antibiotics (penicillin, streptomycin, gentamicin, and amphotericin). AT2 cells reached confluence after 48 h and fluid was removed from the Transwell upper compartment at 72 h to promote formation of a stable air‐liquid interface achieved after a total of 5–6 days.

### 2.8 Human alveolar type II cell influenza infection

AT2 cells in air‐liquid interface culture were infected with PR8 influenza at a multiplicity of infection 1:1 or 10:1 (FFU PR8 to number of AT2 cells on the transwell) in 100 µl total serum‐free DMEM for 90 min. Mock infections were done with DMEM only. Cells were then washed and returned to the incubator for either 24 or 48 h. Supernatants were collected for the measurement of interferon‐λ using an ELISA (R&D Systems). RNA was extracted using Qiagen RNeasy Mini Plus kits, and extract quality and concentration were measured with a DS‐11 Fx+ spectrophotometer. cDNA creation and RT‐PCR were performed as described above, using primers specific for human Ang‐2 (forward: CAGTGGCTAATGAAGCTTGAGAAT; backward: TTTGCTCCGCTGTTTGGTTC) and β‐Actin (forward: TTTTGGCTATACCCTACTGGCA; backward: CTGCACAGTCGTCAGCATATC).

### 2.9 Statistical analyses

Comparisons between two groups were done with unpaired *t*‐test or Mann–Whitney *U* test (when data were not normally distributed). Comparisons of more than two groups were made with ANOVA or Kruskal–Wallis. Repeated measures ANOVA was used for comparisons of multiple groups over more than one time point, and two‐way interaction terms were created for treatment group and time. Log‐rank was used for survival analysis. *p* < 0.05 was considered to be statistically significant. Prism (GraphPad) was used for statistical analyses and graph production.

## 3 RESULTS

### 3.1 Influenza causes dose‐dependent lung injury between 5 and 7 dpi

**FIGURE 1:**
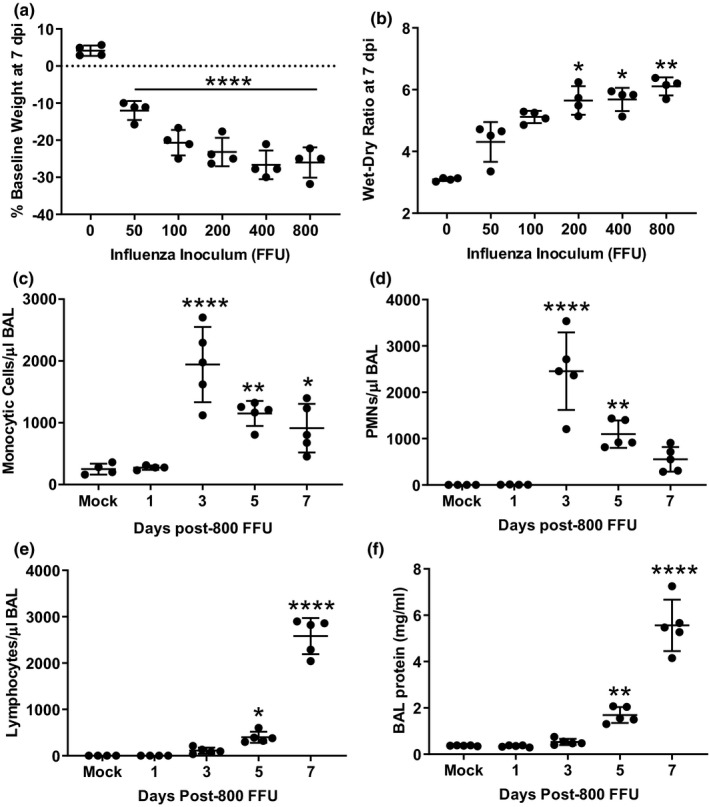
Dose‐dependent evolution of influenza‐induced lung injury over the first week post‐infection. (a) Influenza infection causes dose‐dependent weight loss at 7 dpi. (b) Lung injury, as measured by the wet to dry ratio, increases in a dose‐dependent manner. (c, d) The number of airspace monocytes/macrophages and neutrophils peaks at 3 dpi. (e) In contrast, airspace lymphocytes increase steadily during the first week post‐infection. (f) BAL protein, an indicator of alveolar‐capillary barrier permeability, increases markedly between 5 and 7 dpi. *p* < 0.0001 by ANOVA (a, c–f), *p < 0.05, **p < 0.01, ****p < 0.0001 compared to mock infection by Dunnett's MCT; *p* = 0.002 by Kruskal‐Wallis (b), *p < 0.05, **p < 0.01 compared to mock infection by Dunn's MCT. BAL, bronchoalveolar lavage; MCT, multiple comparisons test

Weight loss (Figure 1a) and pulmonary edema (Figure 1b) at 7 dpi were directly proportional to the inoculum. The dominant inflammatory cell types in the airspaces shifted from monocytes and neutrophils to lymphocytes during the window of greatest lung injury (Figure 1c–e). BAL protein, a marker of alveolar‐capillary barrier permeability, nearly tripled between 5 and 7 dpi (Figure 1f).

### 3.2 Airspace inflammatory cytokines and chemokines parallel the waves of cellular inflammation

**FIGURE 2:**
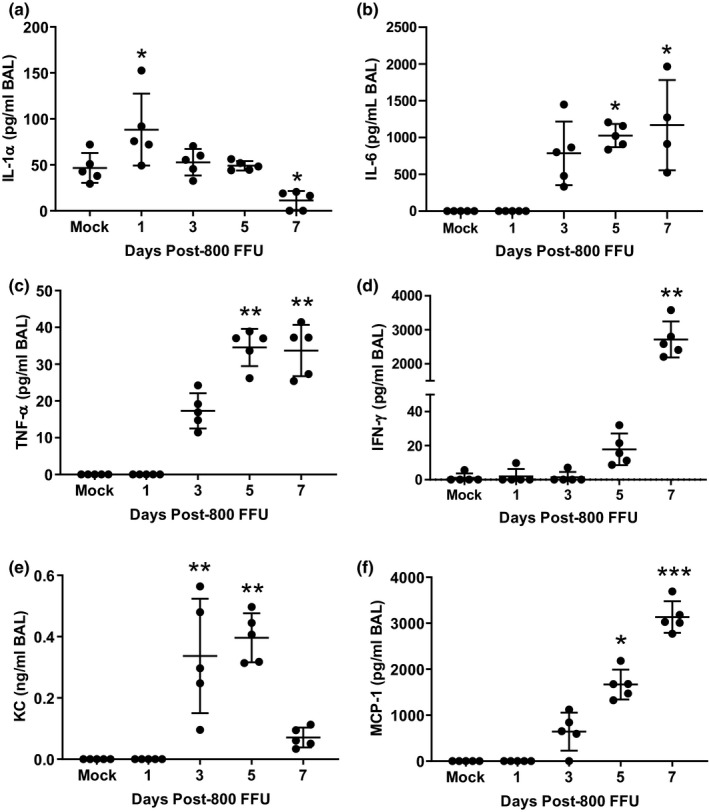
Airspace inflammatory cytokines and chemokines parallel the waves of cellular inflammation. (a) BAL IL‐1α increases at 1 dpi and decreases at 7 dpi compared to mock infection. (b–d) Airspace IL‐6 and TNF‐α are increased by 5 dpi but do not increase during the window of maximum lung injury, whereas IFN‐γ spikes over 100‐fold between 5 and 7 dpi. (e, f) The neutrophil chemokine KC increases early in the first week post‐infection before declining, whereas the monocyte/lymphocyte chemokine MCP‐1 increases steadily. *p* = 0.0003 by ANOVA (a), *p < 0.05 compared to mock infection by Dunnet's MCT; *p* < 0.001 by Kruskal‐Wallis (b–f), *p < 0.05, **p < 0.01, ***p < 0.001 compared to mock infection by Dunn's MCT. BAL, bronchoalveolar lavage; MCT, multiple comparisons test

IL‐1α, an early marker of the pulmonary innate immune response (Dinarello, 2018), increased in the airspaces at 1 dpi (Figure 2a) and declined thereafter, whereas BAL IL‐6 (Figure 2b) and TNF‐α (Figure 2c) increased by 3 dpi and rose at most modestly during the period of rapidly worsening lung injury 5–7 dpi. In contrast, airspace IFN‐γ spiked dramatically between 5 and 7 dpi, in concert with the wave of lymphocytic inflammation (Figure 1e) and increasing alveolar‐capillary barrier permeability (Figure 1f). These patterns of cytokine expression mimic those seen in patients suffering from severe viral illness, as detailed in a recent review (Yuan et al., 2021). As expected, the neutrophil chemokine KC (Figure 2e) and the monocyte/lymphocyte chemokine MCP‐1 (Figure 2f) roughly paralleled the influx of neutrophils, monocytes, and lymphocytes into the airspaces.

### 3.3 Ang‐2 protein increases in the airspaces during the period of greatest lung injury

**FIGURE 3:**
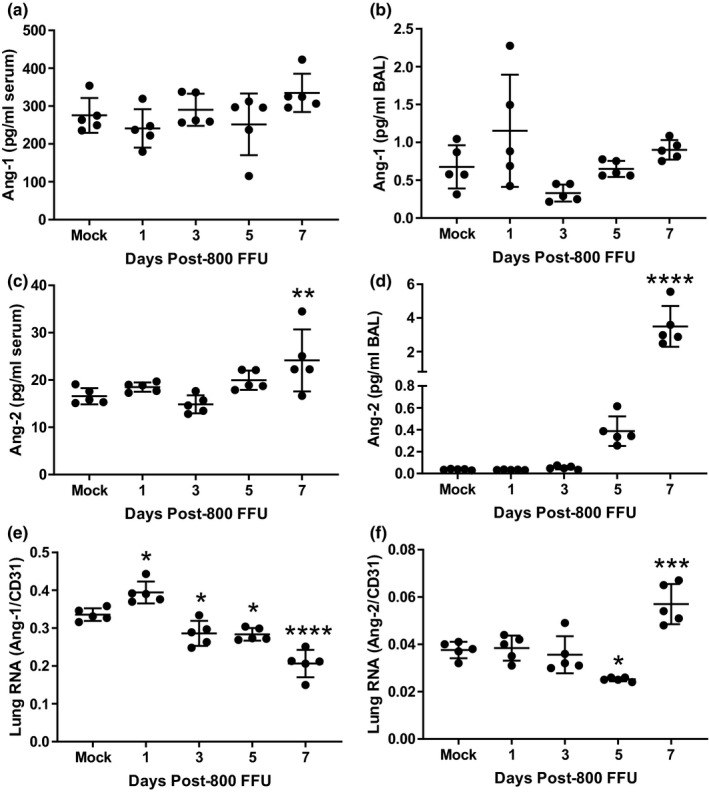
Angiopoietin‐2 protein increases in the airspaces during the period of greatest lung injury. (a, b) Ang‐1 protein levels in the blood and airspaces do not significantly change during the first week post‐infection. (c, d) In contrast, Ang‐2 is modestly increased in the blood at 7 dpi, whereas airspace Ang‐2 increases dramatically between 5 and 7 dpi during the period of greatest lung injury. (e, f) Whole lung Ang‐1 gene expression (normalized to the endothelial marker CD31 to control for the massive inflammatory cell infiltrate) increases modestly 1 dpi and then decreases compared to mock infection between 3 and 7 dpi. Lung Ang‐2 expression remains near mock infection levels until increasing at 7 dpi. (a, b) n.s. by Kruskal‐Wallis; *p* < 0.001 by ANOVA (c–f), *p < 0.05, **p < 0.01, ***p < 0.001, ****p < 0.0001 compared to mock infection by Dunnett's MCT. MCT, multiple comparisons test

Ang‐1 levels in the blood (Figure 3a) and BAL (Figure 3b) were relatively stable throughout the first 7 dpi. Serum levels of Ang‐2 increased modestly 7 dpi compared to mock infection, whereas airspace levels of Ang‐2 increased dramatically between 5 and 7 dpi. Whole lung mRNA for Ang‐1 decreased modestly at 3, 5, and 7 dpi, whereas lung Ang‐2 mRNA increased at 7 dpi (Figure 3f).

### 3.4 Inhibition of Ang‐2 beginning 2 dpi improves oxygenation and survival

**FIGURE 4:**
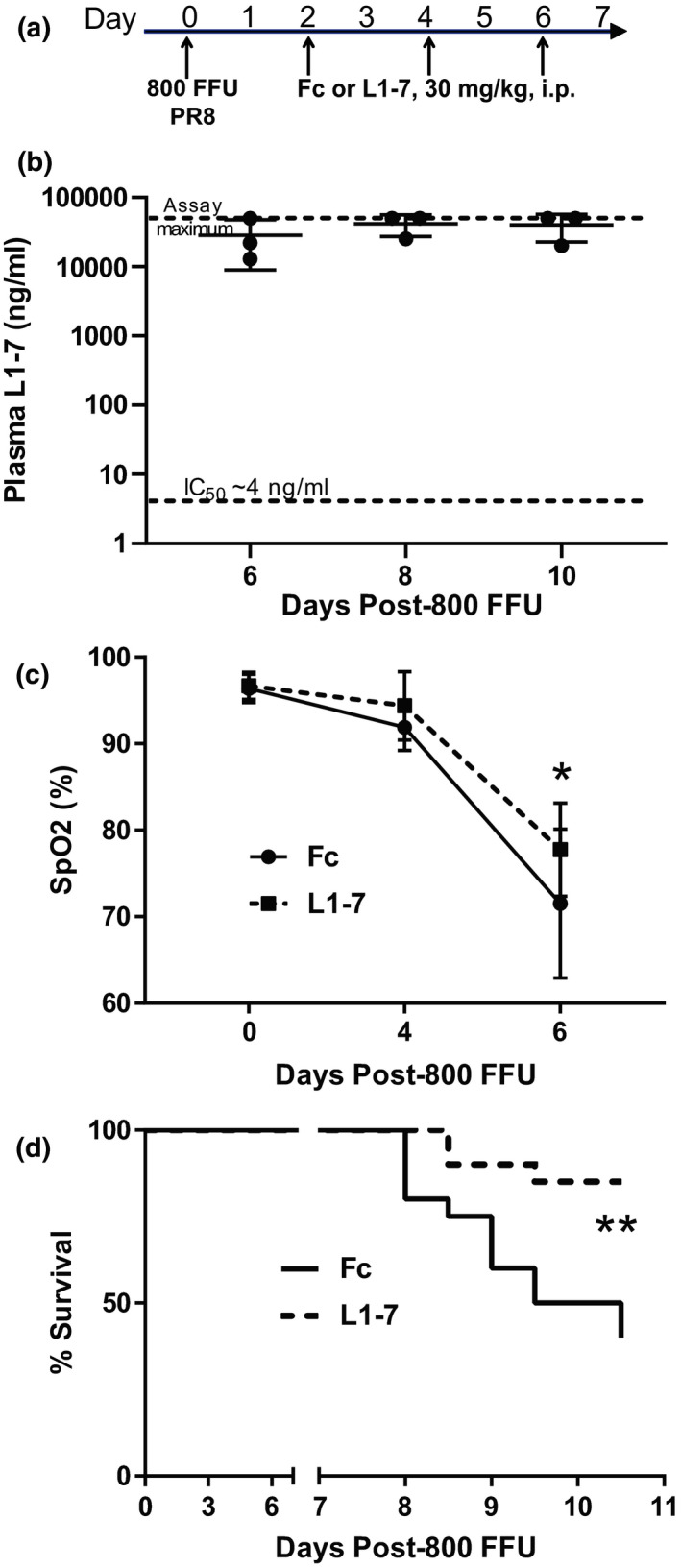
Ang‐2 inhibition improves arterial hypoxemia and survival. (a) Schematic depicting experimental interventions. The Ang‐2 antagonist L1‐7 or control Fc was administered by intraperitoneal injection at 30 mg/kg at 2, 4, and 6 dpi for panels (b–d). (b) A cohort of mice was killed at 6, 8, and 10 dpi and following dosing of Fc or L1‐7 on days 2, 4, and 6. The plasma concentration of L1‐7 at 6 dpi was approximately 2500‐fold higher than the IC_50_ for murine Ang‐2 and this was sustained through 10 dpi. Panels (c, d) are derived from another larger cohort of mice treated with Fc or L1‐7 on days 2, 4, and 6 post‐infection. (c) Decline in arterial oxygen saturation, measured in freely moving mice by cervical oximetry, was significantly attenuated by Ang‐2 antagonism, *n* = 10, *p* = 0.02 for group effect by 2‐way repeated measures ANOVA, *p = 0.01 by Sidak's MCT comparing Fc and L1‐7 at 6 dpi. (d) Ang‐2 antagonism significantly improved survival following high‐dose influenza infection, *n* = 20, ** *p* = 0.004 by Mantel‐Cox. MCT, multiple comparisons test

L1‐7 or a control Human IgG1 Fc was injected at 30 mg/kg, i.p., at 2, 4, and 6 days following inoculation with 800 FFU of PR8 (Figure 4a). This dosing regimen reliably achieved blood levels of L1‐7 above 10 μg/ml (or 170 nM), approximately 2500‐fold higher than the IC_50_ for murine Ang‐2 (0.07 nM or 4.1 ng/ml), at 5, 8, and 10 dpi (Figure 4b). Mice treated with L1‐7 beginning 2 dpi were less hypoxemic (Figure 4c) and had improved survival through 10 dpi (Figure 4d) compared to mice given the control IgG1 Fc.

### 3.5 Ang‐2 inhibition reduced pulmonary edema and alveolar‐capillary barrier permeability without significant effects on inflammation or viral load

**FIGURE 5:**
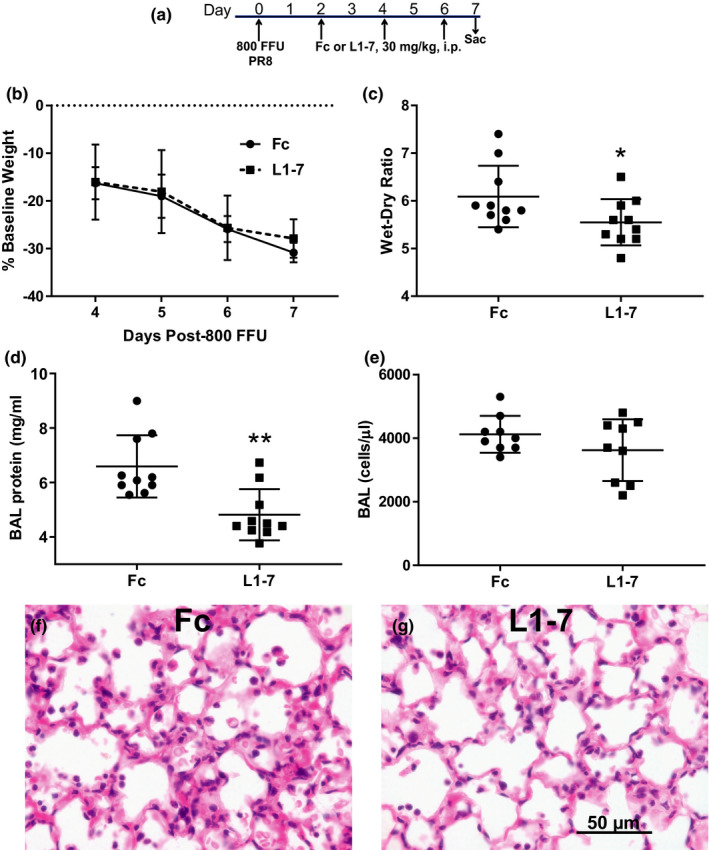
Ang‐2 inhibition improves pulmonary edema and reduces alveolar‐capillary barrier permeability. (a) Schematic depicting experimental interventions. In contrast to Figure 4, all mice in these experiments were killed at 7 dpi. (b) Weight loss was not significantly affected by Ang‐2 antagonism, *n* = 10, *p* < 0.0001 for time effect by 2‐way repeated measures ANOVA. (c) Pulmonary edema measured by wet‐dry ratio was significantly decreased by Ang‐2 antagonism, *p = 0.048 by unpaired *t*‐test. (d, e) BAL protein at 7 dpi was significantly reduced by Ang‐2 antagonism, whereas BAL cellularity was unaffected, **p = 0.003 by Mann–Whitney. (f, g) Representative histological sections at 7 dpi reveal decreased alveolar septal thickening in mice treated with L1‐7 (g) compared to Fc (f), consistent with reduced pulmonary edema. BAL, bronchoalveolar lavage

**FIGURE 6:**
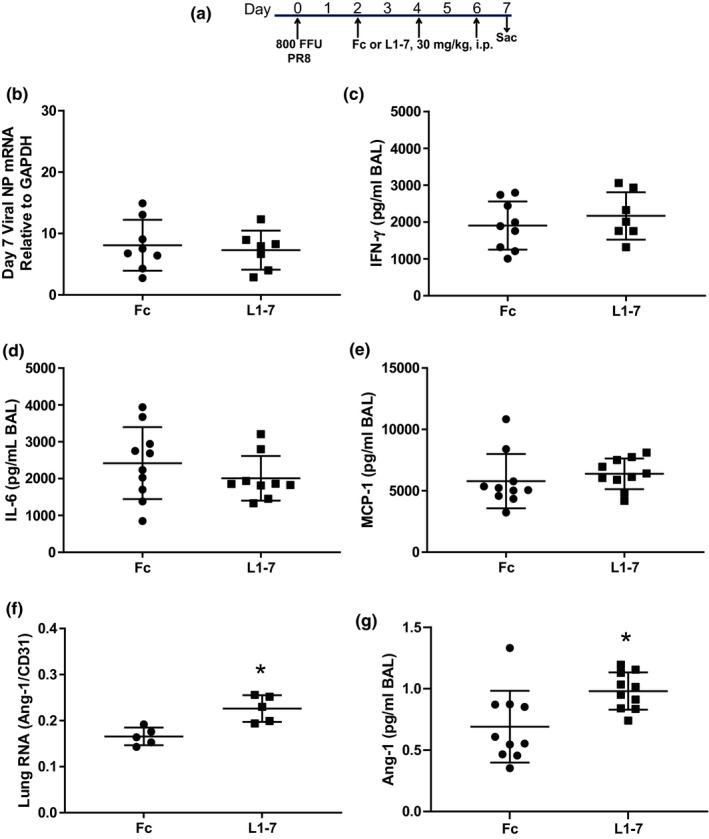
Ang‐2 antagonism does not affect influenza viral load in the lungs nor the levels of relevant inflammatory cytokines in the bronchoalveolar lavage but does increase lung Ang‐1 RNA and protein. (a) Schematic depicting experimental interventions, all mice were killed at 7 dpi. (b) There was no significant difference in influenza viral nucleoprotein (NP) A mRNA in the lung tissue of mice treated with L1‐7 compared to Fc control at 7 dpi. (c–e) Inflammatory cytokines IFN‐γ, IL‐6, and MCP‐1 concentrations in the bronchoalveolar lavage at 7 dpi were not significantly affected by Ang‐2 antagonism with L1‐7. In contrast, L1‐7 treatment increased whole lung Ang‐1 RNA (f) and Ang‐1 airspace protein (g). *p = 0.01 (f) and *p = 0.005 (g) by unpaired *t*‐test

In order to measure lung injury outcomes, mice were treated with L1‐7 or Fc at 2, 4, and 6 dpi with 800 FFU PR8, and then killed 7 dpi. As shown in Figure 5b, there was no significant difference in weight loss which averaged approximately 30% in both groups. Pulmonary edema, measured by the wet to dry ratio, was significantly reduced with L1‐7 treatment (Figure 5c). L1‐7 treatment also significantly reduced BAL protein at 7 dpi, consistent with reduced alveolar‐capillary barrier permeability (Figure 5d). Histological analysis revealed reduced alveolar‐septal thickening in L1‐7 treated mice, consistent with the measured reduction in pulmonary edema (Figure 5f,g). Notably, the total number of BAL cells was not significantly different between L1‐7 and Fc‐treated mice (Figure 5e). Furthermore, there were no differences in the absolute number of neutrophils (median 1025 vs. 856, IQR 1300–595 vs. 1049–614), monocyte/macrophages (median 923 vs. 1103, IQR 1275–708 vs. 1484–978), and lymphocytes (median 1283 vs. 2007, IQR 1980–828 vs. 2258–1454) between L1‐7 and Fc‐treated mice. Similarly, airspace levels of IL‐6, IFN‐γ, and MCP‐1 were not different between Fc and L1‐7 treated mice (Figure 6c–e), nor was viral load (Figure 6b). Interestingly, L1‐7 treatment increased lung RNA and protein for Ang‐1 (Figure 6f,g).

**FIGURE 7:**
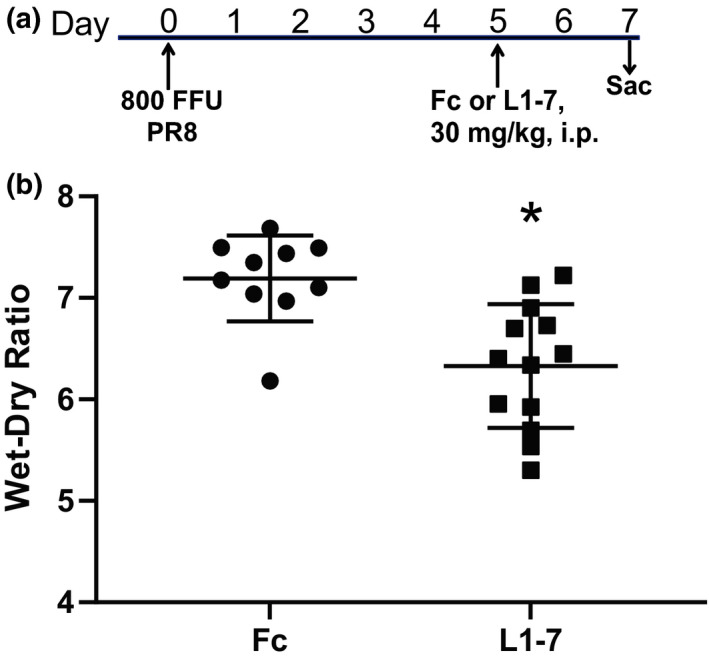
Ang‐2 inhibition on day 5 replicated the therapeutic effect on pulmonary edema. (a) Schematic depicting experimental interventions. (b) L1‐7 treatment delayed until 5 dpi significantly reduced wet‐dry ratio at 7 dpi, *p = 0.001 by unpaired *t*‐test

In order to test whether delaying Ang‐2 antagonism until alveolar‐capillary barrier function is more impaired, we again infected mice with 800 FFU of influenza and then administered a single dose of Fc or L1‐7 at 5 dpi (Figure 7a), a time at which BAL protein is significantly elevated (Figure 1f). As shown in Figure 7b, L1‐7 treatment significantly reduced pulmonary edema relative to Fc control during the critical period between 5 and 7 dpi when alveolar‐capillary barrier function continues to deteriorate rapidly.

### 3.6 Primary human type II alveolar epithelial cells infected with influenza increase Ang‐2 mRNA production

**FIGURE 8:**
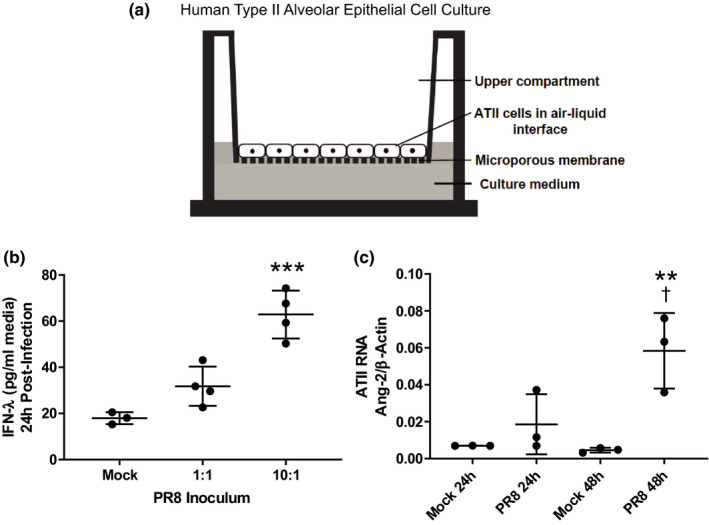
Primary human type II alveolar epithelial cells infected with influenza increase Ang‐2 mRNA production. (a) Schematic depicting primary human alveolar epithelial cell monolayer on Transwell membrane. Cells establish tight junctions and an air‐liquid interface. (b) The type III interferon IFN‐λ in the supernatant of AT‐II cell cultures increased in a dose‐dependent manner 24 h post‐infection with PR8, *p* = 0.0003 by ANOVA, ***p = 0.0002 compared to mock infection by Dunnett's MCT. (c) AT2 cells infected by PR8 increase the expression of Ang‐2 RNA over time, *p* = 0.003 by ANOVA, **p = 0.004 compared to mock 48 h, ^†^ *p* = 0.02 compared to PR8 24 h by Tukey's MCT. MCT, multiple comparisons test

Given the high concentrations of Ang‐2 in the airspaces (Figure 3d), and our prior work reporting that type II alveolar epithelial (AT2) cell monolayers express Tie2 and have reduced protein permeability with Ang‐1 treatment (Rubenfeld et al., 2005), we next tested whether influenza might induce the production of Ang‐2 directly by alveolar epithelial cells. Primary human AT2 cells were isolated from human lungs declined for transplantation as in our previous work (Fang et al., 2010) and were cultured on Transwells until they formed a tight barrier, creating an air‐liquid interface (Figure 8a). Cells were then infected with PR8 influenza at 1:1 or 10:1 (FFU PR8:AT2 cells). As shown in Figure 8b, interferon‐λ, an early type III interferon important in epithelial viral responses (Donnelly & Kotenko, 2010), was induced in a dose‐dependent fashion, demonstrating successful infection. In order to test the possibility that alveolar epithelial cells produce Ang‐2, we harvested AT2 cells after mock infection or 10:1 inoculation with PR8. As shown in Figure 8c, AT2 cells infected with PR8 demonstrated significantly increased Ang‐2 RNA expression compared with mock infection, suggesting that the alveolar epithelium itself may contribute to the high airspace concentrations of Ang‐2 during influenza infection.

## 4 DISCUSSION

Increasing evidence implicates Ang‐2 mediated disruption of Ang‐1/Tie‐2 signaling in the severity of lung injury in critically ill patients. Here we provide evidence that influenza infection in mice increases airspace Ang‐2 levels during the period of greatest lung injury between 5 and 7 dpi. We demonstrate that antagonism of Ang‐2 beginning several days after infection improves oxygenation, pulmonary edema, and survival during severe influenza pneumonia. The lack of major changes in inflammatory cytokine signaling or viral load suggests that Ang‐2 antagonism has a direct beneficial effect on lung barrier function to reduce injury after viral infection as evidenced by improvement in lung edema and BAL protein. Finally, we provide evidence that infected alveolar epithelial cells may be a novel source of Ang‐2. Strengths of this work include (a) temporal characterization of inflammation and ligand expression, (b) intervention after infection and lung injury is established, and (c) the use of clinically relevant endpoints.

Ang‐2 has been mostly well‐studied in patients with sepsis, where its plasma concentration has been shown by multiple investigators to predict pulmonary vascular leak and ARDS (Agrawal et al., 2013; Heijden et al., 2008; Parikh et al., 2006; Reilly et al., 2014). Moreover, Ang‐2 expression is triggered by molecular mediators of sepsis injury like lipopolysaccharide (Mofarrahi et al., 2008) and 2‐chlorofatty acids (Meyer et al., 2017). There is strong evidence in animal models that agonizing Tie2 with Ang‐1 mimetics improves the severity of lung injury in animal models following endotoxin (David et al., 2011; Huang et al., 2008; McCarter et al., 2007) and pneumococcal pneumonia (Gutbier et al., 2017). Similarly, Ang‐2 knockout mice have improved lung injury and survival following hyperoxia (Bhandari et al., 2006). Furthermore, Ang‐2 siRNA (Lomas‐Neira et al., 2014; Stiehl et al., 2014) and blocking antibody (Lomas‐Neira et al., 2016) have been shown to improve lung injury following cecal ligation and puncture.

Viral pneumonia was a major cause of morbidity and mortality even prior to the worldwide pandemic caused by COVID‐19, and the pathways of injury during viral‐mediated lung injury may be distinct from other causes of ARDS (Hendrickson & Matthay, 2013). However, as with bacterial infection, Ang‐2 levels have been reported to be elevated during infection and capillary leak syndromes caused by hantavirus (Nusshag et al., 2017) and dengue virus (Mapalagamage et al., 2020). Furthermore, patients with enterovirus‐71 mediated pulmonary edema have very high levels of Ang‐2 in undiluted pulmonary edema fluid (Qi et al., 2016), similar to prior reports of a mixed population of patients with ARDS (Bhandari et al., 2006). Importantly, a recent study found higher levels of serum Ang‐2 at the time of ICU admission in patients with COVID‐19–related ARDS than in classical ARDS, and Ang‐2 was elevated in COVID‐19 non‐survivors compared to survivors (Spadaro et al., 2021). Higher Ang‐2 in COVID‐19 non‐survivors was corroborated by another study in which plasma Ang‐2 levels at ICU admission predicted mortality in COVID‐19 (Vassiliou et al., 2021).

Ang‐2 is classically thought to be released by smooth muscle and endothelial cells and acts on endothelial cells (Thurston & Daly, 2012). However, Tie2 expression has been shown to be increased in airway epithelial cells during ovalbumin‐induced inflammation, in concert with elevated levels of airspace Ang‐2 (Makinde & Agrawal, 2011). Furthermore, we have previously reported that type II human alveolar epithelial cells express Tie2 and that recombinant Ang‐1 increases Tie2 phosphorylation, decreasing monolayer permeability to albumin (Fang et al., 2010). Importantly, these epithelial monolayer cultures lack lymphatic and endothelial cells, and we have demonstrated that approximately 95% of the cells stain with the AT2 marker HT2‐280 (Fang et al., 2006). The induction of Ang‐2 RNA in human alveolar epithelial cells by influenza infection is a novel finding that deserves future study in influenza and other types of viral pneumonia. Furthermore, the high airspace concentrations of Ang‐2 measured in human clinical samples of undiluted pulmonary edema fluid from patients with ARDS (Bhandari et al., 2006; Qi et al., 2016) and in the current work with influenza suggest that Ang‐2 may have previously unappreciated direct effects on epithelial barrier function, which may act synergistically with increased endothelial permeability to increase alveolar edema.

A major drawback of immunosuppressive therapies such as glucocorticoids during viral pneumonia is impaired pathogen clearance and increased risk for secondary bacterial infection (Ni et al., 2019). Importantly, we found that Ang‐2 inhibition reduced pulmonary edema without causing major changes in the inflammatory molecular and cellular milieu, or lung viral load. A similar dissociation between improved lung injury and unaltered inflammation has been reported with the Ang‐1 mimetic vasculotide during pneumococcal pneumonia (Gutbier et al., 2017) and influenza (Sugiyama et al., 2015). Interestingly, we demonstrate that Ang‐2 inhibition increased lung Ang‐1 RNA and protein levels during influenza‐induced injury. In light of prior work demonstrating the prognostic significance of the Ang‐2 to Ang‐1 ratio in patients with ARDS (Ong et al., 2010) and a number of studies suggesting therapeutic benefit from Ang‐1 agonism (Gutbier et al., 2017; Huang et al., 2008), the increase in Ang‐1 signaling induced by L1‐7 may be an important mechanism of reduced pulmonary edema, particularly given that reduced free Ang‐2 levels likely facilitate Tie2 binding opportunities for Ang‐1.

Limitations of this work include the use of young healthy animals, the lack of later time points to assess longer term outcomes (due to severe weight loss in both groups), and the lack of definitive identification of the cellular source of Ang‐2. However, in the context of other recent studies demonstrating the therapeutic potential of Ang‐1 agonists in influenza pneumonia (Sugiyama et al., 2015), the current work with Ang‐2 antagonism helps build enthusiasm for developing therapeutics targeting this pathway in viral pneumonia, including COVID‐19.

In conclusion, these experimental studies indicated that influenza caused severe lung injury in association with increased lung Ang‐2 RNA and a major increase in Ang‐2 protein in BAL. Inhibition of Ang‐2 improved oxygenation and survival and also reduced pulmonary edema and alveolar‐capillary barrier permeability to protein in association with increased lung Ang‐1 RNA and protein. Influenza also increased the expression of Ang‐2 RNA in human AT2 cells. The increased levels of Ang‐2 in the airspaces during severe influenza pneumonia and the improvement in clinically relevant outcomes after Ang‐2 antagonism indicate that the Ang‐1/Ang‐2 Tie‐2 signaling axis may be a promising therapeutic target in influenza and potentially other causes of viral pneumonia.

## CONFLICT OF INTEREST

This study was partially funded by Amgen. Beyond the named authors, some of whom are employees of Amgen, the sponsor reviewed the manuscript but had no role in study design, data collection and analysis, decision to publish, or preparation of the manuscript. Chun‐Ya Han and Marina Stolina are Amgen stockholders.

## AUTHOR CONTRIBUTIONS

J.G.: conception and design, collection and assembly of data, data analysis and interpretation, and manuscript writing and editing. M.M., L.C., X.F., C.H., and V.C.: collection and assembly of data, data analysis, and interpretation. A.K., T.L., M.S., and M.A.M: data analysis and interpretation, editing, and final approval.
